# Seabuckthorn (*Hippophaë rhamnoides*) Freeze-Dried Powder Protects against High-Fat Diet-Induced Obesity, Lipid Metabolism Disorders by Modulating the Gut Microbiota of Mice

**DOI:** 10.3390/nu12010265

**Published:** 2020-01-20

**Authors:** Caixia Guo, Li Han, Meiping Li, Ligang Yu

**Affiliations:** School of Life Science, Shanxi University, Taiyuan 030006, China; m15503679865@163.com (L.H.); lmpmg@sxu.edu.cn (M.L.); yuligang@sxu.edu.cn (L.Y.)

**Keywords:** seabuckthorn freeze-dried powder, obesity, lipid metabolism disorders, gut microbiota, short-chain fatty acids (SCFAs)

## Abstract

This study aimed to investigate the beneficial effects of seabuckthorn freeze-dried powder on high-fat diet-induced obesity and related lipid metabolism disorders, and further explored if this improvement is associated with gut microbiota. Results showed that seabuckthorn freeze-dried powder administration decreased body weight, Lee’s index, adipose tissue weight, liver weight, and serum lipid levels. Moreover, treatment with seabuckthorn freeze-dried powder effectively reduced fat accumulation by modulating the relative expression of genes involved in lipid metabolism through down-regulation of encoding lipogenic and store genes, including SREBP-1c, PPAR-γ, ACC, and SCD1, and up-regulation of regulating genes of fatty acid oxidation, including HSL, CPT-1, and ACOX. Especially, seabuckthorn freeze-dried powder regulated the composition of gut microbiota, such as increasing the ratio of *Firmicutes*/*Bacteroidetes*, decreasing relative abundance of harmful bacteria (*Desulfovibrio*), and increasing relative abundance of beneficial bacteria (*Akkermansia* and *Bacteroides*). The changes of beneficial bacteria had a positive correlation with genes encoding lipolysis and a negative correlation with genes encoding lipid lipogenesis and store. The harmful bacteria were just the opposite. Besides, changes in gut microbiota had an obvious effect in the secretion of main metabolites—short-chain fatty acids (SCFAs), especially propionic acid. Thus, our results indicated that the seabuckthorn freeze-dried powder could ameliorate high-fat diet-induced obesity and obesity-associated lipid metabolism disorders by changing the composition and structure of gut microbiota.

## 1. Introduction

In recent years, changes in dietary patterns and unhealthy lifestyles have caused an increase in the incidence of chronic diseases [1]. Among these, excessive intake of fat could lead to an imbalance in energy intake and energy expenditure, and this long-term imbalance of energy metabolism could directly contribute to obesity [2]. To date, numerous studies have reported that obesity is a known risk of developing metabolic complications such as type 2 diabetes mellitus, insulin resistance, fatty liver disease, cardiovascular disease, and cancer [3,4]. As a major public health problem, the prevention and treatment of obesity has become a research focus in recent years, and a growing number of literature have reported that dietary modifications, including the use of some plant-derived foods, offered an effective therapy to alleviate obesity and its complications [5].

Generally, excessive dietary fat intake contributes to high circulation of fat in the bloodstream, and it consequently increases adipose tissue and fat accumulation in the liver, leading to lipid metabolism disorders and obesity [6]. Mounting pieces of evidence have found that gut microbiota plays an important role in the development of obesity [6]. Gut microbiota of humans is a complex and neglected organ, containing at least 10^14^ bacteria. Many pieces of literature have verified that gut microbiota alterations affected host metabolism, including nutrient digestion, lipid metabolism, and energy metabolism [7,8], indicating that gut microbiota could be considered as a potential target for the prevention or treatment of obesity and related chronic diseases. In the phylum level, gut microbiota is mainly composed of *Firmicutes*, *Bacteroidetes*, *Verrucomicrobia*, *Saccharibacteria*, *Actinobacteria*, and *Proteobacteria* [9]. Studies have shown that compared with healthy individuals, the proportion of *Bacteroides* decreased and the proportion of *Firmicutes* increased in obese people. Thus, the ratio of *Firmicutes*/*Bacteroides* in the intestine or feces is increased [10,11]. Further, some researchers observed that dietary interventions, especially food crammed with bioactive components, were potential strategies for alleviating obesity and its complications by modulating the gut microbiota [5,12].

Seabuckthorn (*Hippophaë rhamnoides*), as a functional plant homologous to medicine and food, is crammed with a variety of nutrients and biological substances, such as polyphenols, flavonoids, organic acids, vitamin C, polysaccharides, unsaturated fatty acids, and various amino acids required by the human body [13,14]. Although several studies have reported that seabuckthorn extract has the positive effect of lowering plasma cholesterol, increasing intestinal probiotics, improving lipid metabolism enzyme activity, enhancing antioxidant ability, and reducing the incidence of chronic diseases [14,15,16,17], studies using seabuckthorn itself are limited. Seabuckthorn is consumed mainly in the form of whole fruit and related processing products. However, the study of whole food is more meaningful by considering the loss of nutritional components during processing. Seabuckthorn freeze-dried powder is made by low-temperature freeze-drying technology, which retains all useful nutrients such as peel, pulp, fruit oil, and seed oil, and still has strong biological efficacy. Thus, this study investigated the effect of seabuckthorn freeze-dried powder as a dietary intervention on obesity and obesity-associated lipid metabolism disorders in high-fat diet mice. Furthermore, we aimed to investigate if those beneficial effects of seabuckthorn freeze-dried powder are associated with the modulation of gut microbiota.

## 2. Materials and Methods 

### 2.1. Materials and Diets

Seabuckthorn freeze-dried powder (24% energy value, 2001 KJ/100 g) was purchased from Urumqi Desert Kangpin Trading Co., Ltd. (Urumqi, China). The primary ingredients of seabuckthorn freeze-dried powder are shown in Table 1. Normal diet (D12450B, 10% kcal fat, 70% kcal carbohydrate, 20% kcal protein, 3.85 kcal/g) and high-fat diet (D12492, 60% kcal fat, 20% kcal carbohydrate, 20% kcal protein, 5.24 kcal/g) were prepared by Beijing Keao Cooperation Feed Co., Ltd. (Beijing, China), and the production license number was SCXK (JING) 2014-0010. 

### 2.2. Animals Experimental Design and Dietary Treatment 

A total of 30 six-week-old male C57BL/6 mice (18.3 ± 2.0 g) were purchased from Cavens Experimental Animals Co., Ltd. (Suzhou, China; license number: SYXK (SU) 2016-0011). These mice were housed individually under temperature-controlled environment conditions: temperature 23.0 ± 3.0 °C, humidity 55.0% ± 5.0%, and a standard 12-h light/black cycle with free access to food and water. All the animal studies were conformed to the principles for laboratory animal research outlined by the Animal Welfare Act (Pub. L. 89–544). The animal protocol used in this study was approved by the Institutional Animal Care and Use Committee of Shanxi University (No. SXULL2019060). After acclimation for two weeks, the abnormal mice were knockout and the rest were randomly allocated into three groups (*n* = 8): normal control diet (NC), high-fat diet (HFD), or a high-fat diet united by gavaging the homogenate of seabuckthorn freeze-dried powder (HSFP). According to relevant animal toxicology experiments and the human–mouse conversion coefficient, the mice of the HSFP group were given 4.0 mg/ (g.d. body weight) seabuckthorn freeze-dried powder by oral gavage [18]. Besides, the mice in NC and HFD groups received an equal volume of physiological saline, and all the mice were fed via intragastric gavage for 10 weeks. Meanwhile, the food intake and the condition of mice were monitored every day, and the body weight was monitored weekly.

### 2.3. Sample Collection

At the end of the experiment, the mice were fasted for 12 h and anesthetized by a subcutaneous injection of 1% pentobarbital sodium solution. After deep anesthetic, the blood sample was collected by orbital sinus to the anticoagulant tube and then centrifuged at 3000 rpm for 10 min at 4 °C to gather serum for biochemical analysis. Then, the liver and the white adipose tissue (subcutaneous adipose tissue, epididymal adipose tissue, and perinephric adipose tissue) were removed and weighed. A small part of the liver and epididymal adipose tissue was fixed in 10% neutral formaldehyde solution for histopathology observation, and the rest of the tissues were quickly frozen in liquid nitrogen and stored at −80 °C until analysis. During the last experiment period, the feces were collected in sterile EP tubes and stored at −80 °C for the following gut microbiota and metabolites analysis, respectively.

### 2.4. Biochemical Analysis of Serum Parameters

The serum parameters including total cholesterol (TC), triglyceride (TG), high-density lipoprotein cholesterol (HDL-C), and low-density lipoprotein cholesterol (LDL-C) were quantified using the assay kits, which were purchased from Nanjing Jiancheng Bioengineering Institute (Nanjing, China). All operations were performed according to the manufacturer’s instructions. 

### 2.5. Histological Analysis of Liver and Adipose Tissues

Fixed liver and adipose tissues were embedded in paraffin and sectioned at 5–7 µm thickness by tissue slicer (Leica RM2235, Shanghai Ouqi Electronic Technology Co. Ltd., Shanghai, China). After that, sections were stained with hematoxylin–eosin (H&E), and pathological morphology was observed using an Olympus CX31 microscope (Olympus Corporation Ltd., Tokyo, Japan). Then, the average areas of adipose tissue were measured by using Axio Vision Real 4.6 software to statistic five different visual fields of each sample.

### 2.6. Gene Expression Analysis

The liver and adipose tissue of the three groups were, respectively, homogenized in Trizol reagent, after which the total RNA for measuring real-time quantitative PCR was isolated by using the RNA Simple Total RNA Kit (TIANGEN Biochemical Technology Co., Ltd., Beijing, China). Meanwhile, RNA quality and quantity were assessed by the ratios of 260 nm/280 nm. Then, complementary DNA (cDNA) was synthesized by reverse transcription from 1 μg total RNA using the First-Strand cDNA Synthesis kit (US Everbright Inc., Suzhou, China). Then, relative mRNA expression was quantified by using the SYBR Green qPCR Master Mix kit (US Everbright Inc., Suzhou, China) on the real-time fluorescent quantitative PCR instrument, ABI Fast 7500 (Applied Biosystems, California, USA). The relative expression levels of each gene were normalized according to the $2^{-\Delta \Delta t}$ method using *β*-actin as a reference gene. The primer information of sequences is shown in Table 2 and was provided by GenScript (Nanjing) Co., Ltd. (Nanjing, China).

### 2.7. Gut Microbiota Analysis 

Fecal samples collected in the cecum were sent to Biomarker Technologies Co., Ltd. (Beijing, China) to analyze the composition and structure of the gut microbiota. The bacterial genomic DNA of fecal samples was extracted using the PowerSoil^®^ DNA Isolation Kit (Beijing, China). The V3–V4 region of the 16S rDNA genes were resigned by a polymerase chain reaction. PCR amplification was performed and thermal cycling conditions were as follows: an initial denaturation at 95 °C for 5 min, followed by 15 cycles at 95 °C for 1 min; 50 °C for 1 min; and 72 °C for 1 min, with a final extension at 72 °C for 7 min, maintained at 4 °C). The specific bacterial primers were the forward primer 5′-ACTCCTACGGGAGGCAGCA-3′ and the reverse primer 5′-GGACTACHVGGGTWTCTAAT-3′. After the purification of the target gene, the qualified DNA was further amplified then paired-end sequenced (2 × 250) on the Illumina MiSeq platform at Biomarker Technologies Co., Ltd. (Beijing, China). The analysis of raw paired-end reads from the original DNA sequence data was performed using BMK Cloud (www.biocloud.net). 

### 2.8. Gut Metabolites Analysis by Gas Chromatography (GC)

The standards of acetic acid, propionic acid, and butyric acid were purchased from Rhawn Chemical Technology Co., Ltd. (Shanghai, China). Firstly, the standard acids configured different concentrations were detected to prepare standard curves. Two hundred milligrams of fecal samples were dissolved with 1.3 mL methanol and 1.0 mL distilled water in the 5 mL EP tubes and centrifuged at 4500 rpm for 10 min. Then, the supernatant was taken 0.1 mL 50% concentrated sulfuric acid was added, placed for 1 h at 70 °C. Then, 1 mL ether was added until the mixture cooled to room temperature. The final mixture was centrifuged at 13,000 rpm for 5 min, and the supernatant was brought out for GC analysis [19]. The chromatographic separation was performed on an FFAP capillary column using helium (1.0 mL/min) as a carrier gas. One microliter of sample solution was injected with 15:1 of split ratio, and the injector temperature was 250 °C. The temperature produces were performed with the following protocol: initial temperature 100 °C for 1 min; elevated at 3 °C/min to 130 °C for 1 min; elevated at 20 °C/min to 200 °C for 3 min [19,20]. According to the standard curves, we could calculate the relative content of each acid finally. 

### 2.9. Statistical Analysis

All experimental data were presented as mean ± standard deviation (SD) and analyzed by one-way ANOVA followed by Duncan’s multiple range test using SPSS 20.0 (Beijing Stats Data Mining, Beijing, China) to evaluate the significant difference between the groups and *p* < 0.05 was considered the statistically significant difference. Correlation analysis (R) was performed using the Spearman coefficient in accordance with normal distribution and outlier.

## 3. Results

### 3.1. Seabuckthorn Freeze-Dried Powder Alleviated Obesity in High-Fat Diet-Induced Obese Mice

**Figure 1:**
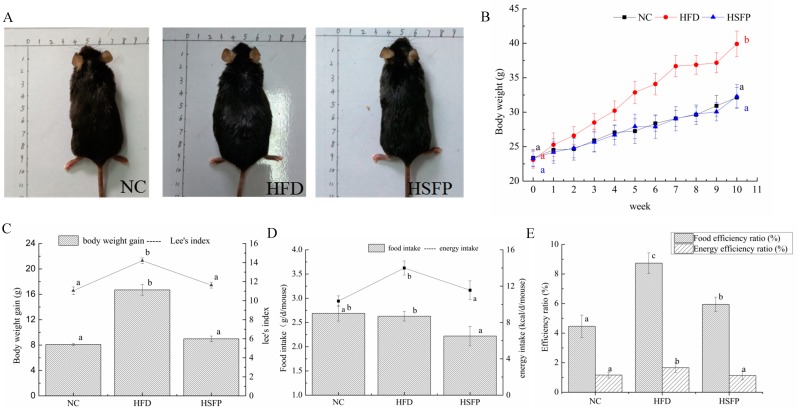
Effects of seabuckthorn freeze-dried powder on the feature indexes of obesity in C57BL/6 obesity mice reduced by the high-fat diet. NC: the mice of normal diet, HFD: the mice of high-fat diet, HSFP: the mice of high-fat diet united by gavaging the homogenate of seabuckthorn freeze-dried powder. (**A**) Body size of representative mice. (**B**) Growth curve of mice. (**C**) Body weight gain and Lee’s index (Lee’s index = body weight∗1000body length3. (**D**) Food intake and energy intake. (**E**) Food efficiency ratio: (weight gain/food intake for the whole experiment period) × 100) and energy efficiency ratio: (weight gain/energy intake for the entire experiment period) × 100). In our study, all data were expressed as means ± SD, graph bars with different letters on the top correspond to statistically significant results (*p* < 0.05) based on one-way ANOVA analysis followed by Duncan’s multiple range test.

**Figure 2:**
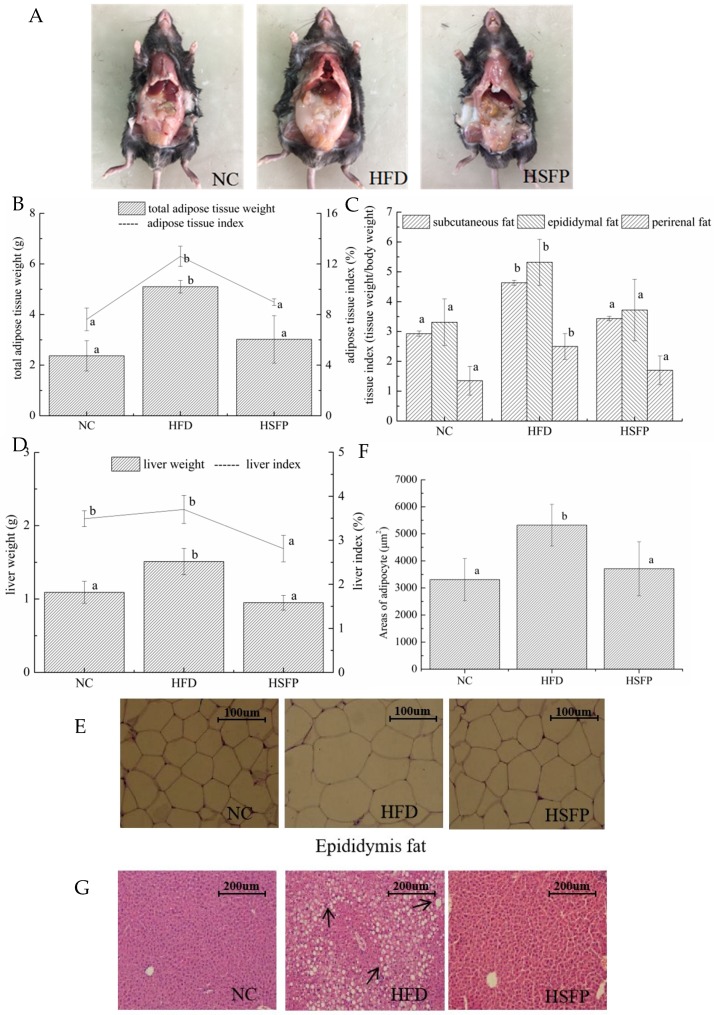
Seabuckthorn freeze-dried powder supplementation attenuates high-fat diet-induced fat accumulation, adipocyte hypertrophy, and hepatic steatosis. (**A**) Anatomy, (**B**,**C**) total adipose tissue weight, and three parts adipose tissue weight. (**D**) Liver weight. (**E**) H&E staining of epididymal white adipose tissue sections (scale: 100 μm, photographed at 400× magnification) and (**F**) average area of adipocyte. (**G**) H&E staining of liver sections (scale: 200 μm, photographed at 200× magnification). In our study, all data are expressed as means ± SD. Graph bars with different letters on the top correspond to statistically significant results (*p* < 0.05) based on one-way ANOVA analysis followed by Duncan’s multiple range test.

At the beginning of the experiment, the initial body weight of mice was not significantly different in all groups (*p* > 0.05), but the mice of the HFD group had a significant increase of body weight at the final stage of the experiment (*p* < 0.05) (Figure 1A,B). Furthermore, Lee’s index of the HFD group was higher than that of the NC group (*p* < 0.05). The mice in the HSFP group significantly decreased body weight gain and Lee’s index (*p* < 0.05) compared to the HFD group (Figure 1C), suggesting that seabuckthorn freeze-dried powder treatment could prevent the obesity induced by the high-fat diet. Meanwhile, the food intake of mice in each group was tracked regularly, showing that the food intake of the HSFP group mice was lower than the NC and HFD groups (Figure 1D). The energy intake of the HSFP was lower than the HFD group (*p* < 0.05); however, no difference was observed in energy intake between the NC and HSFP groups (*p* > 0.05). The supplement of seabuckthorn freeze-dried powder significantly decreased the food efficiency ratio and energy efficiency ratio compared with the HFD group (*p* < 0.05) (Figure 1E). We can also observe that HFD feeding for 10 weeks led to a significant increase in liver weight compared with the NC group (*p* < 0.05), and seabuckthorn freeze-dried powder treatment controlled the increase induced by the high-fat diet (Figure 2D). One of the most important features of the gained body weight is the deposition of adipose tissue. Therefore, we weighted the white adipose tissues, including subcutaneous fat, epididymal fat, and perirenal fat. It can be seen from Figure 2C that the weight of three adipose tissues in the HFD group exhibited an obvious increase compared with the NC group (*p* < 0.05), and after supplementing with freeze-dried seabuckthorn, the mass was decreased. Especially, the change of epididymal fat was most significant by showing an increase of 1.160 g in the HFD group compared with the NC group.

### 3.2. Pathological Alterations of Adipose Tissue and Liver

The pathological alterations in epididymal adipocyte and liver in all groups are shown in Figure 2E,G. The adipocyte cells of the NC group were arranged in order and all sizes were almost the same, while the adipocyte size in the HFD group was inconsistent and aggravated. Meanwhile, Figure 2F shows that the adipocyte areas in the HFD group were significantly elevated to approximately 5320.26 μm^2^, which was significantly bigger than that in the NC group (3310.43 μm^2^) (*p* < 0.05). Seabuckthorn freeze-dried powder administration generated a beneficial effect that the adipocyte area in epididymal adipose tissue was reduced to approximately 3710.56 μm^2^ in the HSFP group, which was not significantly different from that of the NC group (*p* > 0.05). Therefore, we concluded that seabuckthorn freeze-dried powder intervention could alleviate adipocyte hyperplasia and hypertrophy. 

Moreover, excessive fat intake could not only result in the accumulation and lesion of adipose tissue but also cause the disorder of lipid metabolism in the liver further to hepatic steatosis. From the perspective of the H&E staining for liver tissue, we can observe that lipid droplets in hepatocytes were significantly increased in the HFD group compared with the NC group (*p* < 0.05). In contrast, the hepatocytes of the mice in the HSFP group hardly showed lipid droplets, suggesting that seabuckthorn freeze-dried powder supplement effectively inhibited excessive lipid accumulation.

### 3.3. Seabuckthorn Freeze-Dried Powder Improved Metabolic Disorders in High-Fat Diet-Induced Obese Mice 

**Figure 3:**
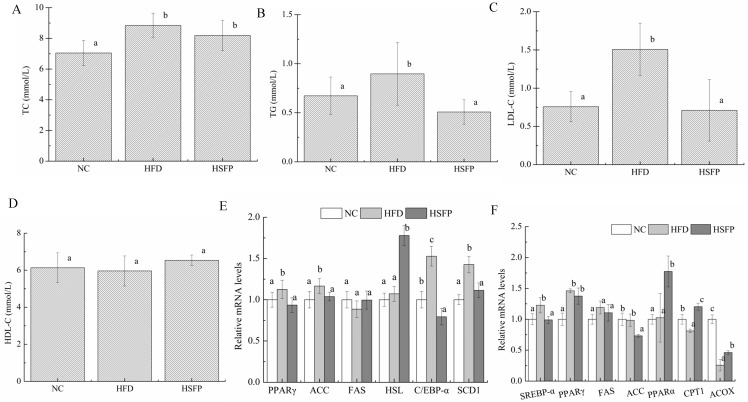
Seabuckthorn freeze-dried powder regulated lipid metabolism. (**A**–**D**) were serum lipid indexes, including total cholesterol (TC), triglyceride (TG), low-density lipoprotein cholesterol (LDL-C), and high-density lipoprotein cholesterol (HDL-C), and the expression levels of genes related to lipid synthesis and oxidation of the epididymal white adipose tissue (**E**) and liver (**F**), including peroxisome proliferator-activated receptor-γ (PPAR-γ), acety1-CoA carboxylase (ACC), fatty acid syntheses (FAS), sterol regulatory element-bingding protein-1c (SREBP-1c), peroxisome proliferator-activated receptor-α (PPAR-α), stearoyl-CoA desaturase 1 (SCD1), CCAAT/enhancer-binding protein alpha (C/EBP-α), hormone-sensitive triglyceride lipase (HSL), carnitine palmitoyl transferase-1 (CPT-1), peroxisomal acyl coenzyme a oxidase (ACOX). All data are shown as means ± SD. Graph bars with different letters on the top correspond to statistically significant results (*p* < 0.05) based on one-way ANOVA analysis followed by Duncan’s multiple range test.

As far as we know, excessive fat accumulation often accompanies metabolic disorder, so the levels of serum TC, TG, LDL-C, and HDL-C were detected (Figure 3A–D). Although the levels of serum TC, TG, and LDL-C of the HFD group were higher than that of the NC group, the supplement of seabuckthorn freeze-dried powder in the HSFP had a significant reduction of the serum TG and LDL-C levels compared with HFD group (*p* < 0.05). On the contrary, the HDL-C level did not differ among the three groups (*p* > 0.05).

### 3.4. Seabuckthorn Freeze-Dried Powder Regulated the Expression of Obesity-Associated Genes in the Mice

The gene expression of adipogenesis and lipogenesis was closely correlated with the lipid accumulation in the adipose tissue and liver. Therefore, we quantified the mRNA expression levels of metabolism-related genes in the epididymal adipose tissue and in the liver to investigate the potential molecular mechanisms.

As shown in Figure 3E, the expression levels of the genes in the epididymal adipose tissue, including peroxisome proliferator-activated receptor-γ (PPAR-γ), acety1-CoA carboxylase (ACC), stearoyl-CoA desaturase 1 (SCD1), and CCAAT/enhancer-binding protein alpha (C/EBP-α), were significantly elevated under the high-fat diet feeding (*p* < 0.05). Supplementation of seabuckthorn freeze-dried powder could down-regulate the expression of PPAR-γ, ACC, and SCD1 compared to the HFD group, and showed no significant differences compared to NC mice (*p* > 0.05). However, seabuckthorn freeze-dried powder treatment did not alter the expression of C/EBP-α as compared with the HFD group. Although no significant difference in the expression of hormone-sensitive triglyceride lipase (HSL) was observed between the NC group and the HFD group (*p* > 0.05), seabuckthorn freeze-dried powder treatment enhanced that. Meanwhile, it can be seen from Figure 3F that the hepatic genes expressions of sterol regulatory element-binding protein 1c (SREBP-1c), PPAR-γ, and ACC in the HFD group were increased relative to the NC group, but the change of ACC was tiny (*p* > 0.05). Compared to the HFD group, the mice in the HSFP group exhibited significant down-regulation of SREBP-1c and ACC expression (*p* < 0.05). Notably, the genes expressions of PPAR-α, carnitine palmitoyl transferase-1(CPT-1), and peroxisomal acyl coenzyme a oxidase (ACOX) in the liver were tested, which promote energy expenditure and fatty acid oxidation. The results showed that the levels of CPT-1 and ACOX expression in the HFD group were lower (*p* < 0.05) than the NC group (Figure 3F), whereas the expression of PPAR-α had no significant difference between HFD group and NC group. In addition, we observed significant up-regulation of those by seabuckthorn freeze-dried powder treatment. Besides, the negligible change of fatty acid syntheses (FAS) expression was observed in three groups, either in adipose tissue or liver.

### 3.5. Seabuckthorn Freeze-Dried Powder Altered the Composition and Structure of the Gut Microbiota 

**Figure 4:**
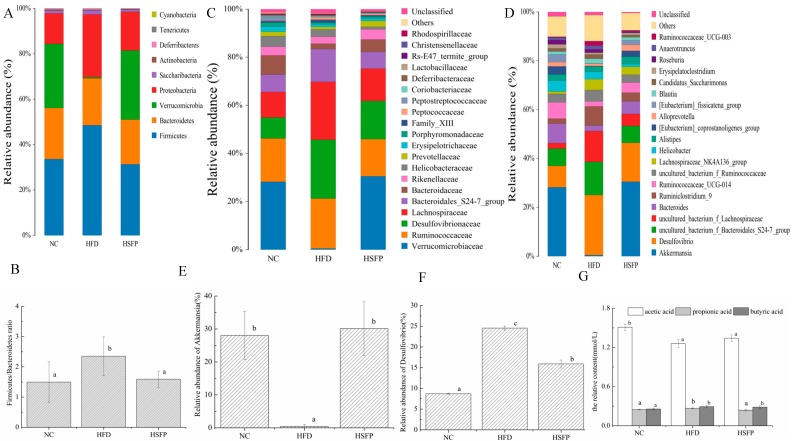
Seabuckthorn freeze-dried powder administration alters the composition and structure of gut microbiota. The composition and proportion of bacterial communities at the phylum (**A**,**B**), family (**C**) , and genus (**D**) level. Specific genus of bacteria (**E**); *Akkermansia* (**F**); *Desulfovibrio* (**G**). The relative abundance of short-chain fatty acids (SCFAs) in three groups. Data were shown as means ± SD. Graph bars with different letters on the top correspond to statistically significant results (*p* < 0.05) based on one-way ANOVA analysis followed by Duncan’s multiple range test.

To identify the compositional differences on gut microbiota by the effect of seabuckthorn freeze-dried powder, the relative abundance at phylum, family, and genus level was investigated, and the results are shown in Figure 4. At the phylum level (Figure 4A), *Firmicutes*, *Bacteroidetes*, *Verrucomicrobia*, and *Proteobacteria*, as a common phylum, were observed in all groups, and their relative abundance reached more than 90%. Based on the relative abundance, the content of *Firmicutes* and *Proteobacteria* had a significant increase in the HFD group compared to the NC group (*p* < 0.05), and seabuckthorn freeze-dried powder supplementation fully prevented the increase of those. As far as the content of *Bacteroidetes*, there was no significant difference among the three groups (*p* > 0.05). While the increase ratio of *Firmicutes*/*Bacteroidetes* induced by a high-fat diet was effectively prevented during the seabuckthorn freeze-dried powder administrated period (Figure 4B), apart from this, seabuckthorn freeze-dried powder intervention resulted in a dramatic decrease in the content of *Verrucomicrobia* compared to the HFD group. At family level (Figure 4C), seabuckthorn freeze-dried powder supplementation suppressed the obvious increases in the relative abundance of *Lachnospiraceae* (HFD: 24.12%; HSFP: 13.44%), *Ruminococcaceae* (HFD: 20.68%; HSFP: 15.42%), *Bacteroidales-S24-7-group* (HFD: 13.57%; HSFP: 6.95%), and *Desulfovibrionaceae* (HFD: 24.52%; HSFP: 15.91%) induced by a high-fat diet. Meanwhile, seabuckthorn freeze-dried powder prevented the decreases in the relative abundance of *Erysipelotrichaceae* (HFD: 1.07%, HSFP: 1.06%), *Prevotellaceae* (HFD: 1.01%, HSFP: 2.442%), *Bacteroidaceae* (HFD: 2.22%, HSFP: 5.13%), *Verrucomicrobiaceae* (HFD: 0.52%; HSFP: 30.49%), and *Rikenellaceae* (HFD: 2.91%, HSFP: 4.25%) compared with the HFD group. Especially, the change of *Verrucomicrobiaceae* was most obvious, which is similar to the relative abundance in the NC group (NC: 28.24%; HSFP: 30.49%). Based on the top 20 genera with relative abundance greater than 1%, the gut bacteria relative abundance was shown in Figure 4D. The changing trend of bacteria was similar to those observed at the phylum and family levels. Notably, in the HFD group (Figure 4E,F), we noticed that the relative abundance of *Akkermansia* was extremely lower (0.51%) than the NC group (28.23%), and *Desulfovibrio* (24.51%) was extremely higher compared with the NC group (8.72%), while those were normalized to the level of the NC group after the seabuckthorn freeze-dried powder intervention (30.49% and 15.49%, respectively). We also explored a detailed analysis of other obesity-related intestinal bacteria in mice. In the HFD group, a dramatic decrease in the relative abundance of *Bacteroides*, *Roseburia*, *Alistipes*, *Erysipelatoclostridium*, *Helicobacter*, *Alloprveotella*, and *Ruminococcaceae-UGC-014*, and a significant increase of the *Blautia*, *Lachnospiraceae-NK4A136-group*, *Ruminiclostridium-9*, *Anaeotruncus*, *Ruminiclostridium-UCG-03*, and *Candidatus-Saccharimonas* were observed compared with the NC group. Especially, *Intestinimonas*, Oscillibacter, and *Lachnolostridium* were observed only in the HFD group, reflecting that they have been likely to a positive correlation with obesity. 

**Figure 5:**
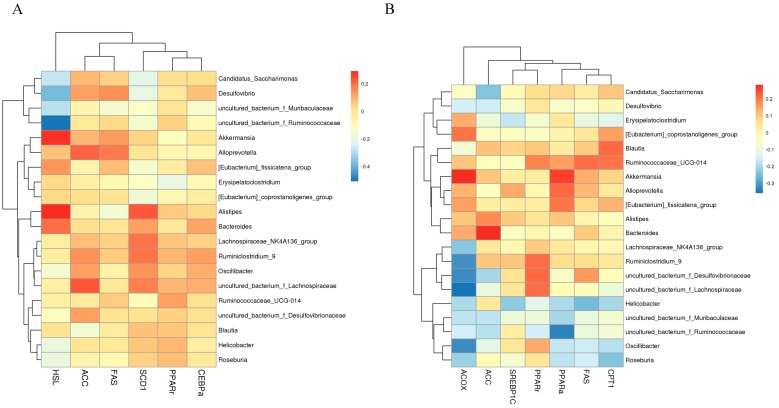
Spearman’s correlation between the metabolic genes and the relative abundance top 20 bacteria at the genus level (**A**) metabolic genes in the adipose tissue; (**B**) metabolic genes in liver tissue. The different colors of bar represented the degree of association (red, positive correlation; blue, negative correlation).

To determine whether the changes of obesity-related genes expression in adipose and hepatic tissues were associated with changes in the specific bacterial taxa at the genus level of mice consuming seabuckthorn freeze-dried powder, we used Spearman’s correlation to analysis. The results of adipose tissue were shown in Figure 5A, the abundance of *Akkermansia*, *Alistipes,* and *Bacteroides* was positively correlated with that of HSL expression, and the abundance of *Desulfovibrio* and *Candiatus_Saccharimonas* were negatively correlated with HSL expression. For some bacteria that only exist in the HFD group, *Oscillibacter* and uncultured bacterium_f_*Lachnospiraceae* had a positive correlation with the relative expression of ACC and SCD1, and *Oscillibacter* had a negative correlation with the relative expression of HSL. In the liver (Figure 5B), the abundance of *Akkermansia* was positively correlated with the mRNA expression levels of PPARα and ACOX. The abundance of *Desulfovibrio* was negatively correlated with mRNA expression levels of ACC. Meanwhile, uncultured bacterium_f_*Lachnospiraceae* and *Oscillibacter* had a positive correlation with the relative expression of PPARγ, and a negative correlation with the relative expression of PPARα, CPT1, and ACOX. The phenomenon of correlation suggested that the beneficial bacteria were positively correlated with the genes encoding decomposition, and the harmful bacteria were negatively correlated with the synthesized genes.

### 3.6. The Effect of Seabuckthorn Freeze-Dried Powder on Gut Metabolites

The gut metabolites, mainly short-chain fatty acids (SCFAs) including acetic acid, propionic acid, and butyric acid, were investigated by GS analysis, and the results are shown in Figure 4G. Compared with the mice of the NC group, the content of acetic acid was decreased significantly (*p* < 0.05), whereas propionic acid and butyric acid increased with significant difference (*p* < 0.05) in the HFD group. The contents of propionic acid and butyric acid were much lower than the content of acetic acid. After seabuckthorn freeze-dried powder intervention, the contents of acetic acid and propionic acid were reverted and approached to the NC group to a certain extent. Collectively, the SCFAs in the HFD group had an obvious decrease compared with the NC group and seabuckthorn freeze-dried powder treatment could prevent the trend.

## 4. Discussion

Obesity is defined as the accumulation of excess adipose tissue, accompanying the increase of body weight and fat deposition in the liver, causing corresponding metabolism dysfunction. Accumulating evidence showed that the daily consumption of foods rich in bioactive compounds, such as fruits and vegetables, could confer a beneficial effect on anti-obesity and obesity-association metabolism diseases [21]. In our study, the results showed that daily supplementation with seabuckthorn freeze-dried powder at 4.0 mg/(g.d. body weight) could significantly decrease body weight, Lee’s index, adipose tissue weight, liver weight, and other obesity-related indexes. Meanwhile, we also observed that seabuckthorn freeze-dried powder administration dramatically prevented the increase of serum TC, TG, and LDL-C induced by a high-fat diet. So, we concluded that a certain amount of intake of seabuckthorn freeze-dried powder could prevent obesity and obesity-related lipid metabolism disorders caused by a high-fat diet. Nevertheless, we also observed that the food intake and energy intake of the HSFP group were significantly lower than the HFD group. To determine whether weight loss is associated with reduced food intake, we calculated the food efficiency ratio and the energy efficiency ratio, which represent the relationship between weight gain and food intake. We found that even if they eat the same high calorie fat-rich foods, seabuckthorn intake could not only reduce food intake to prevent the development of obesity, but also reduce the utilization efficiency of food and energy. Similar results were obtained in the dietary intervention of *Phaseolus vulgaris* extract, fresh angelica *keiskei* juice, and mulberry ethanol extract on obesity induced by a high-fat diet [12,21,22].

Generally, the excessive accumulation of fat in the body is closely related to lipid metabolism, including fat synthesis, mobilization, and decomposition [23]. The liver and adipose tissue, as main metabolism places, has been noticed by a lot of researchers. After feeding, fat enters the small intestine where it is hydrolyzed and emulsified into small fat droplets combined with bile salts. Triglycerides are digested to form fatty acids and glycerol, among which fatty acids partly are absorbed by the intestinal mucosa, and the rest could be resynthesized into triglycerides. They then form into chylomicrons with proteins, cholesterol, and phospholipids. Formed chylomicrons are transported to tissue through the lymphatic system and blood where they are catalyzed by lipoprotein lipase to fatty acids and glycerol in the capillaries of the tissues during the stage. Then, the fatty acids enter the liver, and they are used as substrates for *β*-oxidation or resynthesis of triglycerides. It is known that hepatic de-novo lipogenesis and fatty acid oxidation are the primary metabolic pathways that regulate hepatic lipid metabolism [24]. During the metabolism, SREBP-1c and PPARα were key regulators in lipogenesis and fatty acid oxidation pathways, respectively [25]. SREBP-1c, a transcription factor, is a critical regulator that stimulates fatty acid and triglyceride biosynthesis through up-regulation of rate-limiting enzymes, such as FAS and ACC [26]. ACC, as rate-limiting enzymes in the first step of the fatty acid synthesis, could catalyze Acetyl-CoA convert to malonyl-CoA, then Acetyl-CoA and malonyl-CoA were synthesized to a long-chain fatty acid under the expression of SREBP-1c and FAS [1]. PPARγ, as a transcription factor in adipogenesis, is involved in the accumulation of lipids droplets [27]. In the present study, we observed that seabuckthorn freeze-dried powder supplement inhibited lipid lipogenesis in the liver by down-regulating the over-expression of SREBP-1c, PPARγ, and ACC induced by a high-fat diet. In addition, the catabolism of fatty acids is mainly fatty acid *β*-oxidation [28]. PPARα, a ligand-activated nuclear hormone receptor, stimulates fatty acid oxidation by up-regulating downstream genes, such as ACOX and CPT1 [29]. While, seabuckthorn freeze-dried powder could enhance the expressions of PPARα, CPT1, and ACOX to speed up the oxidation of lipids. The process of lipid consumption begins with the activation of fatty acids into fatty acyl-CoA (ACOX), and the fatty acyl-CoA come into the mitochondria to *β*-oxidation under sufficient conditions of oxygen supply. ACOX is the rate-limiting enzyme of peroxidase fatty acid *β*-oxidation, and CPT1 is the rate-limiting enzyme of *β*-oxidation of fatty acids, which is determined as the speed of fatty acid oxidation [30]. In terms of research about preventing or improving obesity and obesity-related diseases, it was noted that supplementation with polyphenols, polysaccharides, or flavonoid-rich plants could ameliorate obesity symptoms by activating fatty acid *β*-oxidation or increasing lipid metabolism [21]. For example, quercetin, resveratrol, kaempferol glycosides, and polysaccharide reduced the body weight gain as well as the accumulation of adipose tissue by down-regulating PPARγ and SREBP-1c expression and up-regulating PPARα expression. [6,31]. Our research confirmed that seabuckthorn freeze-dried powder could alleviate obesity-induced abnormal lipid metabolism, and it might be related to abundant bioactive substances like polyphenols, flavonoids, and polysaccharides.

Excess energy is stored in the form of triacylglycerols in adipose tissue. In this study, the significant increase in adipose tissue weight in the HFD group was observed. After the treatment of seabuckthorn freeze-dried powder, the adipose tissue weight significantly reduced, especially epididymal fat. In the fat synthesis period, except for PPAR-γ, FAS, and ACC genes, SCD1 also worked. SCD1 is a key regulatory gene that catalyzes the synthesis of monounsaturated fatty acids, and the expression of SCD1 is frequently used to characterize lipid deposition in adipose tissue. C/EBP-α, a transcription factor, is encoding for lipid differentiation [32]. Treatment of seabuckthorn freeze-dried powder could down-regulate the over-expression of PPAR-γ, ACC, and SCD1 induced by a high-fat diet, but no significant effect was observed on the expression of C/EBP-α compared with the HFD group. In addition to fat synthesis and storage, lipolysis also occurs in adipose tissue owing to the homeostasis mechanism of body metabolism. HSL, as a key rate-limiting enzyme in the decomposition of triglycerides in adipose tissue, is mainly expressed in adipose tissue, which can catalyze stored TG into free acids and glycerol, thereby improving animal fat abnormal deposition. After seabuckthorn freeze-dried powder intervention, the expression of HSL had a significant increase compared with the HFD group. Based on these results, we concluded that seabuckthorn freeze-dried powder plays a vital role in reducing fat accumulation and losing weight by suppressing the expression of synthetic and store genes or enhancing the expression of decomposition genes. Anti-obesity effects of seabuckthorn freeze-dried powder could result from polyphenols, total sugar, total acid, or dietary fiber [33]. Recent animal studies reported that some plants cramming with bioactive ingredients, such as bamboo-shaving, angelica *keiskei*, and citrus peel, reduced body weight gain, Lee’s index, and ameliorated lipid metabolic disorders by altering the composition and structure of gut microbiota [6,21,34]. On this basis, we further investigated whether the beneficial effect of seabuckthorn freeze-dried powder on the improvement of obesity was associated with the changes of gut microbiota. In the phylum level, compared with the normal individual, high-fat diet consumption generally leads to an increase of the *Firmicutes* proportion or a decrease of the *Bacteroidetes* proportion [35]. That is to say, the ratio of *Firmicutes* and *Bacteroidetes* is associated with obesity and obesity-related metabolic syndrome. Our results showed that the daily consumption of seabuckthorn freeze-dried powder had a significant decrease in the ratio of *Firmicutes* and *Bacteroidetes*. Moreover, the specific bacteria, such as *Bacteroidales-S24-7-group*, *Lachnospiraceae*, and *Verrucomicrobiaceae*, are associated with specific biological effects [36]. In our case, we observed that the relative abundance of *Verrucomicrobiaceae* decreased in high-fat diet mice. *Bacteroidales-S24-7-group* and *Lachnospiraceae* increased by feeding with a high-fat diet, which is inconsistent with previous reports [36]. After seabuckthorn freeze-dried powder intervention, *Verrucomicrobiaceae* was significantly enriched, and the relative abundance of *Bacteroidales-S24-7-group* and *Lachnospiraceae* was restored to the NC group, suggesting that dietary seabuckthorn freeze-dried powder has a beneficial effect on reversing the relative abundance of beneficial bacteria to the normal state. In addition, *Desulfovibrionaceae*, a harmful bacterium producing LPS, was significantly enhanced in high-fat diet mice [36]. After seabuckthorn freeze-dried powder supplementation, the level of *Desulfovibrionaceae* exhibited a significant decrease. Talking specifically about the genus, *Akkermansia*, *Bacteroides*, and *Ruminococcaceae_UGC_014*, which were reported that the relative abundance of those was negatively correlated with obesity-related indices, indicating that they may play an important role in the prevention of obesity [21], among which *Akkermansia*, the only cultivated intestinal representative of the *Verrucomicrobia* phylum, was negatively associated with the metabolic disorders in many clinical and preclinical studies [35,37,38]. Some studies reported that dietary or drug-enriched *Akkermansia* could protect mice from high-fat diet-induced obesity. In our study, seabuckthorn freeze-dried powder administration could enhance the abundance of beneficial bacteria, including *Akkermansia*, *Bacteroides,* and *Ruminococcaceae_UGC_014*, and decrease the abundance of harmful bacteria, such as *Desulfovibrio* and *Erysipelatoclostridium*. Because the total relative abundance of *Akkermansia* and *Desulfovibrio* accounted for more than 40%, far higher than other bacteria, they played a vital role in anti-obesity effects. We further investigated the direct association between genera and lipid metabolism genes. Spearman’s correlation analysis revealed that the abundance of *Akkermansia* had a significantly positive correlation with genes (PPARα, ACOX, and HSL) encoding lipolysis and a negative correlation with that of lipogenic genes (PPARγ). On the contrary, the abundance of *Desulfovibrio* showed a positive correlation with lipogenic genes (FAS and ACC), but lipolytic genes (HSL, PPARα, ACOX, and CPT1) showed a negative correlation. These data were consistent with the previous reports that dietary can regulate the metabolism genes expression and the relative abundance of beneficial bacteria and harmful bacteria [39]. All bacteria interacted and worked together to change abnormal fat accumulation in the host health, after which the composition and structure of gut microbiota might affect the expression of genes associated with obesity. 

Admittedly, it should not be overlooked that changes of gut microbiota also affect the main metabolites - SCFAs, including acetic acid, propionic acid, and butyric acid. A large number of studies had confirmed that obesity up-regulated the metabolic pathway that generates SCFAs to improve the expression level, which is associated with excessive energy intake in obese individuals [40]. In return, SCFAs could regulate processes related to obesity directly or indirectly. Among these, acetic acid and propionic acid were reported that they affect appetite to reduce food intake. Beyond these, some studies demonstrated that butyric acid, mainly produced by fermentation of *Bacteroidetes* and *Clostridium*, can reduce intestinal motility, promote nutrient absorption, and lead to obesity and complications due to excessive energy storage of the host [41]. In our study, we discovered that seabuckthorn freeze-dried powder intervention could reverse the increase of propionic acid induced by a high-fat diet and decrease butyric acid level. In addition, the total content of the acid was higher in the HSFP group than in the HFD group, suggesting that seabuckthorn freeze-dried powder may decrease food intake and food effective ratio to suppress the fat accumulation, further to ameliorate obesity and its complications. 

## 5. Conclusions

In summary, we have demonstrated that a dietary choice of supplementation with 4 mg/(g.d. body weight) seabuckthorn freeze-dried powder has a positive effect on preventing body weight gain, adipose tissue mass increase, and improving serum lipids, hepatic lipids accumulation and hepatic steatosis in high-fat induced obesity mice. These observations might be related to metabolic gene expression changes in fat accumulation and the changes in gut microbiota. Our results demonstrated that seabuckthorn freeze-dried powder suppressed fat accretion by down-regulating lipid synthesis and accumulation genes and up-regulating lipolytic genes. In addition, seabuckthorn freeze-dried powder improved the abundance of some gut bacteria, such as showing the lower ratio of *Firmicutes*/*Bacteroidetes*, a higher relative abundance of *Akkermansia*, and lower relative abundance of *Desulfovibrio*, among which the abundance of *Akkermansia* was a positive correlation with lipidolysis genes and was negative with lipid synthesis and accumulation genes. The abundance of *Desulfovibrio* was just the opposite. Furthermore, changes in gut microbiota also affected the main metabolites, such as acetic acid, propionic acid, and butyric acid. 

Above all, seabuckthorn freeze-dried could ameliorate obesity and obesity-associated lipid metabolism disorders induced by the high-fat diet through modulation of gut microbiota. So, seabuckthorn freeze-dried powder may be used for ameliorating obesity and obesity-related metabolic syndrome as potential for prebiotics or functional food. Nevertheless, our study remains with some shortcomings. We elucidate the associated metabolism regulated by seabuckthorn freeze-dried powder on the metabolic level and gut microbiota, but a more detailed mechanism remains to be explored. 

## Figures and Tables

**Table 1:** The primary ingredients in seabuckthorn freeze-dried powder.

Ingredient	Content
Total polyphenols (mg/g)	6.55 ± 0.29
Total sugar (g/100 g)	22.83 ± 0.44
Total acid (malic acid as a standard) %	14.38 ± 0.62
Soluble solid (°Brix)	62.54 ± 5.30
Soluble dietary fiber (g/100 g)	26.15 ± 0.53
Insoluble dietary fiber (g/100 g)	1.51 ± 0.10
Vitamin C (mg/100 g)	23.24 ± 0.51

Notes: All data are presented as mean ± standard deviation (SD). Total polyphenols content was measured by using the Folin–Ciocalteu colorimetric method. Total sugar was determined by the direct titrimetric method with Fehling’s reagent. Total acid was assayed using the titrimetric method. The content of the soluble solid (°Brix) was determined using the refractometric method by a portable refractometer (BM-FG103; Shanghai BM Optical Instruments Manufacture Company, Ltd., Shanghai, China). The contents of soluble dietary fiber and insoluble dietary fiber were recommended by an enzymatic gravimetric method. Vitamin C content was determined following the Chinese National Standard.

**Table 2:** Primer sequences of detected genes.

Gene	Primer Sequences		Tm	Sequence Number	Product PCR (bp)
PPARγ	Forward	TTTTCAAGGGTGCCAGTTTCGATCC	61	NM_001127330.1	198
	Reverse	AATCCTTGGCCCTCTGAGAT	58		
ACC	Forward	GGCCAGTGCTATGCTGAGAT	59	NM_133360.2	108
	Reverse	AGGGTCAAGTGCTGCTCCA	59		
FAS	Forward	GCTGCGGAAACTTCAGGAAAT	57	NM_007988.3	84
	Reverse	AGAGACGTGTCACTCCTGGACTT	61		
SREBP-1c	Forward	CACAGCGGTTTTGAACGACA	58	NM_011480.3	147
	Reverse	CTCTCAGGAGAGTTGGCACC	58		
HSL	Forward	CTGGAACTAAGTGGACGCAAG	56	NM_001039507	91
	Reverse	CAGACACACTCCTGCGCATAGAC	60		
C/EBP-α	Forward	TCGGTGCGTCTAAGATGAGG	57	NM_001287523	179
	Reverse	TCAAGGCACATTTTTGCTCC	55		
SCD1	Forward	AGAGAACTGGAGACGGGAGT	57	NM_009127	130
	Reverse	AACACCCCGATAGCAATATCCA	57		
PPARα	Forward	CAAGGCCTCAGGGTACCACT	59	NM_001113418.1	111
	Reverse	TTGCAGCTCCGATCACACTT	58		
CPT-1	Forward	CTCAGTGGGAGCGACTCTTCA	59	NM_013495.2	105
	Reverse	GGCCTCTGTGGTACACGACAA	60		
ACOX	Forward	GCCTTTGTTGTCCCTATCCG	58	NM_001271898.1	189
	Reverse	TACATACGTGCCGTCAGGC	58		
*β*-actin	Forward	CAGGCATTGCTGACAGGATG	58	NM_007393	156
	Reverse	TGCTGATCCACATCTGCTGG	58		
